# Modified Lipoproteins in Diabetic Retinopathy: A Local Action in the Retina

**DOI:** 10.4172/2155-9570.1000314

**Published:** 2013-12-18

**Authors:** Jeremy Y Yu, Timothy J Lyons

**Affiliations:** 1 Centre for Experimental Medicine, School of Medicine, Dentistry and Biomedical Science, Queen’s University Belfast, Northern Ireland, UK; 2 Harold Hamm Diabetes Center and Section of Endocrinology and Diabetes, University of Oklahoma Health Sciences Center, Oklahoma City, OK, USA

**Keywords:** Blood retina barrier, Diabetic retinopathy, Dyslipidemia, Fenofibrate, Lipoprotein, Oxidized LDL, Pericytes

## Abstract

Clinical epidemiological studies have revealed relatively weak, yet statistically significant, associations between dyslipidemia/dyslipoproteinemia and diabetic retinopathy (DR). Recent large interventional studies, however, demonstrated an unexpectedly robust efficacy of fenofibrate on the development of DR, possibly independent of plasma lipids. To unify the apparent discrepancies, we hypothesize that plasma lipoproteins play an indirect but important role in DR, contingent on the integrity of the blood-retina-barrier (BRB). In retinas with an intact BRB, plasma lipoproteins may be largely irrelevant; however, important effects become operative after the BRB is impaired in diabetes, leading to lipoprotein extravasation and subsequent modification, hence toxicity to the neighbouring retinal cells. In this hypothesis, BRB leakage is the key, plasma lipoprotein concentrations mainly modulate its consequences, and fenofibrate has intra-retinal actions. This review summarizes our current knowledge of the direct effects and mechanisms of modified lipoproteins on retinal cells and their potential contribution to the pathogenesis of DR.

## Clinical, Epidemiological Evidence Supports an Indirect, yet Important, Role for Dyslipidemia in DR

The role of dyslipidemia/dyslipoproteinemia in diabetic retinopathy (DR) has been a matter of debate, but the weak associations between plasma lipid levels and DR status have dampened interest. Many earlier studies explored the relationship between circulating levels of lipids and lipoproteins and the severity of DR, either cross-sectionally or longitudinally [1-17]. In general, these revealed correlations between retinopathy and standard measures of plasma cholesterol, including total and LDL cholesterol, and LDL-to-HDL cholesterol ratio. This work has been previously reviewed in detail [18-22], and some recent important studies are summarized below.

The Pittsburgh Epidemiology of Diabetes Complications study [23], a prospective study with 657 type 1 diabetic patients, showed that concentrations of serum triglycerides, and to a lesser extent LDL cholesterol, were associated with retinopathy. Higher levels of LDL cholesterol and triglycerides were associated with progression to proliferative diabetic retinopathy (PDR). In the Early Treatment Diabetic Retinopathy Study (ETDRS), serum lipid levels were measured in 2709 patients [24]: those with elevated total or LDL cholesterol levels at baseline were twice as likely to have retinal hard exudates as those with normal levels. The Hoorn study [25], a population-based cross-sectional study with 2484 diabetic and non-diabetic individuals, found that the prevalence of DR was positively associated with serum cholesterol and triglyceride levels, and that retinal hard exudates were associated with elevated total and LDL cholesterol. In the Atherosclerosis Risk In Communities study [26], the presence of retinal hard exudates was correlated with LDL cholesterol and lipoprotein (a). With the aid of improved lipoprotein fractionation technology, we evaluated the relationship of plasma lipoproteins with DR in more detail in a Diabetes Control and Complications Trial (DCCT) sub-cohort of 988 type 1 diabetic patients (440 women and 548 men) [27]. Lipoproteins were measured by conventional lipid profile and nuclear magnetic resonance lipoprotein subclass profile (NMR-LSP), and in addition, apolipoprotein A1 (apoA1), apoB, lipoprotein (a), and susceptibility of LDL to oxidation were determined. Conventional profiles showed that the severity of retinopathy was positively associated with triglycerides and negatively with HDL cholesterol. NMR-LSP measures identified retinopathy as being associated with small and medium VLDL and negatively with VLDL size. In male subjects only, retinopathy was positively associated with small LDL, LDL particle concentration, apoB concentration, and small HDL, and negatively associated with large LDL, LDL size, large HDL, and HDL size. The findings were consistent with a role for dyslipoproteinemia in the pathogenesis of DR. Most recently, in a cross-sectional study of 224 type 1 and type 2 diabetic patients, apoA1 (inverse association), apoB and apoB-to-apoA1 ratio (positive associations) were significantly and independently associated with DR and its severity [28]. Serum apolipoprotein levels were believed to be stronger biomarkers for DR than the traditional lipid measures in that study [28].

Overall, a prominent conclusion of most of the epidemiological studies is the positive association between plasma LDL (i.e. levels of apoB and cholesterol, or particle size) and DR. However, this association, although of statistical significance, is only moderate in magnitude, and not of sufficient strength to be useful in defining a patient’s individual risk for DR. A further consideration is that, without diabetes, dyslipidemia does not appear to cause retinal disease, and native LDL even at higher concentrations does not pose significant toxicity to cultured retinal cells.

Besides quantitative lipid measures, qualitative changes of lipoproteins such as formation of oxidized LDL (ox-LDL; for a detailed review refer to [29]), a well-established risk factor for atherosclerosis [30-32], have also been associated with retinopathy. A small but significant amount of ox-LDL (ranging from 0.001% in healthy people to 5% of total LDL in disease states [33]) was detectable in plasma, and was elevated significantly in diabetes [29]. In the Diabetes Control and Complications Trial/Epidemiology of Diabetes Interventions and Complications (DCCT/EDIC) cohort, we showed that increased circulating levels of AGE-LDL- and ox-LDL-immune complexes were associated with higher risk of severe non-proliferative retinopathy (NPDR) and PDR in type 1 diabetes over many years [34]. In this cohort, ox-LDL-immune complexes were also associated with the progression of carotid intima-media thickness [35] and coronary calcification [36]. In type 2 diabetes, it has been reported that patients affected by retinopathy had higher levels of IgG autoantibodies against malondialdehyde-modified apoB-100 in their circulation [37], and the authors also found that higher levels of IgG specific for the native apoB-100 fragments p45 and p210 were associated with DR, but appeared to be protective of coronary disease progression [37]. The reason for such an apparent difference between micro- and macro-vascular complications is unclear and needs to be further elucidated. Overall, the data support a role for modified lipoproteins in the pathogenesis of DR.

Interest in the role of lipids and lipoproteins has been amplified recently by the results of two large prospective studies of type 2 diabetes patients, Action to Control Cardiovascular Risk in Diabetes (ACCORD) [38] and the Fenofibrate Intervention and Event Lowering in Diabetes (FIELD) Study [39], which demonstrated unexpected yet robust benefits of fenofibrate, a drug that has been used to reduce elevated plasma triglycerides, on DR. In the ACCORD study, after 4 years, fenofibrate reduced the rate of DR progression (6.5% vs. 10.2% with placebo) by at least three steps on the ETDRS Severity Scale in patients who were also receiving simvastatin. In the FIELD study, fenofibrate reduced the frequency of laser treatment for diabetic macular edema (DME) by 31% and for PDR by 30%. Interestingly, however, its effect was not clearly attributable to the systemic lipid-lowering effects[39], suggesting that the mechanisms could be unrelated to the drug’s effects on plasma lipids, and/or could be related to tissue lipid processing that is not readily reflected in systemic circulation: i.e. fenofibrate may act through intra-retinal pleiotropic effects. In this regard, fenofibrate has been reported to decrease plasma ox-LDL [40], modulate the lectin-like ox-LDL receptor 1 (LOX-1, scavenger receptor for ox-LDL) [41], and attenuate cellular effects of ox-LDL [42]. We have shown that, in diabetic animal models, intravitreal fenofibrate attenuated angiogenic and inflammatory responses via the PPARα receptor [43]. In addition, mechanisms independent of PPARα receptor have been reported for fenofibrate [44,45], and may contribute to the attenuation of lipotoxicity in retinal pericytes [46]. The findings with fenofibrate were not entirely without precedent: many years ago, another fibrate drug, clofibrate [47], and more recently etofibrate [48], were also shown to have beneficial effects on DR. Of interest, ‘statins’ which are generally more effective than fibrates in preventing cardiovascular events, seem to be less beneficial than fibrates in DR: they have, however, been shown to reduce retinal hard exudates [49].

## A Unifying Hypothesis for Lipoproteins in DR

**Figure 1:**
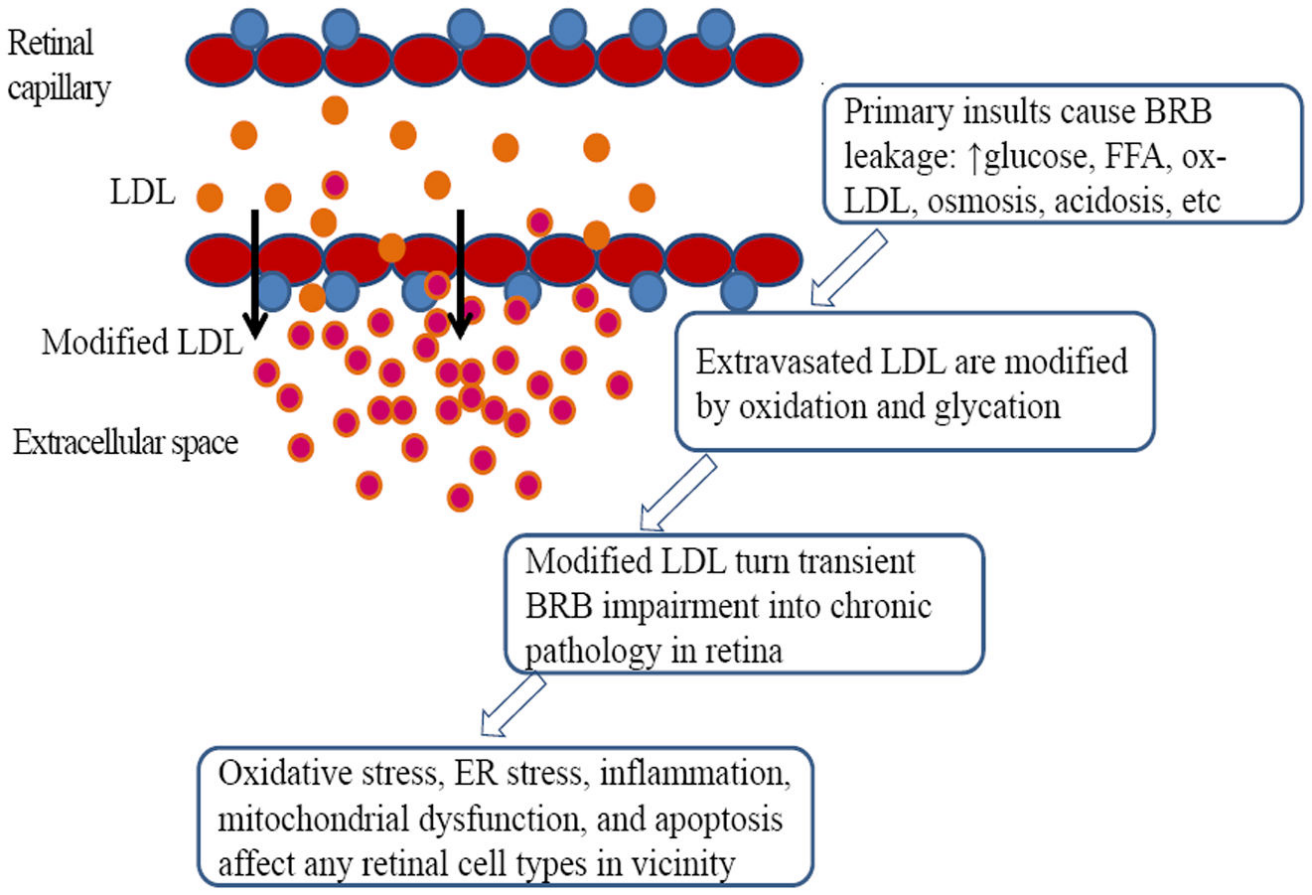
A working hypothesis of modified lipoproteins in the pathogenesis of DR. The role of circulating lipoproteins in DR depends on the integrity of BRB. Normally, plasma LDL does not cause retinal damage, but plasma ox- LDL (mostly mildly modified) may contribute to the initial BRB impairment, together with many other metabolic factors that are commonly seen in diabetes. Once the BRB becomes leaky, even in a short period, LDL can extravasate, aggregate, and become progressively modified by oxidation and glycation in the extracellular milieu, resulting in generalized damages to all retinal cell types in proximity. Extravasation of lipoproteins is expected to gradually turn intermittent, transient BRB impairment into a prolonged, chronic pathological state. In this model, fenofibrate may attenuate retinopathy by modulating intra-retinal lipid processing and inflammation, with the efficacy unrelated to its systemic lipid-lowering effect. The retinal pathology caused by extravascular modified lipoproteins is largely isolated from the circulating lipids, consistent with the generally weak association between plasma lipids and DR in epidemiological studies.

To provide a working model that will connect the apparently disparate observations (i.e. relatively weak association data from epidemiological studies, robust efficacy of fenofibrate in clinical intervention studies, and extensive laboratory data showing deleterious effects of modified, but not native, lipoproteins on retinal cells (discussed below)), our evolved thinking is that plasma lipoproteins play a ‘hidden’, indirect role on DR, which is dependent on the breakdown of the blood-retina-barrier (BRB) (Figure 1). In normal retina with an intact BRB, plasma lipoproteins are largely irrelevant; however, their effects become operative after the BRB becomes deficient (as in diabetes), allowing extravasation of lipoproteins which then become modified (i.e. oxidized and/or glycated) in tissue, rendering them toxic towards nearby retinal cells. In this hypothesis, BRB leakage is the key, and plasma lipoprotein concentrations simply modulate its consequences. One limitation of the model is that the action of lipoproteins occurs only as a secondary effect of BRB leakage, not as the primary initiator. BRB impairment may be caused by many common, intermittent metabolic stresses that are present in diabetes, such as high and fluctuating glucose, free fatty acids, oxidative stress and osmotic stress [50-54], all of which may be acutely exacerbated during episodes of ketoacidosis. Extravasation of lipoproteins, we suggest, can gradually turn a transitory BRB impairment into prolonged, chronic pathology. Also, because of their cytotoxic effects on retinal capillary cells, higher levels of ox-LDL in circulation may pose a direct noxious effect on the BRB [55-57], contributing to the initiation of damage. Overall, the role of ox-LDL in DR is essentially analogous to that in atherosclerosis, in which elevated plasma levels of LDL and modified LDL are associated with cardiovascular disease, where the modification of LDL and its harmful effects occur primarily in the arterial intima, not in plasma. In the retina, certain unique features are operative: retinal lipoprotein exudates appear in the perivascular extracellular space adjacent to the neural retina, due to the small size of retinal capillaries [49], and may thus produce generalized retinal neurovascular injuries [58]. Also, because LDL is normally excluded completely from the retina, the ‘fold increase’ once BRB leakage occurs is much greater in the retina than in the arterial intima.

Consistent with this model for DR, Benarous et al. [59] recently proposed that serum lipids were involved in the late-stage, severe form of DME, through lipoprotein exudation following BRB breakdown. In a prospective cohort of 500 type 1 and type 2 diabetic patients, they reported that serum lipids were independently associated with the clinically significant macular edema only, but not with DR, or with mild or moderate DME. Indeed, since DME occurs after BRB breakdown, dyslipidemia may be more of a risk factor for DME than for DR [60]. We suggest that LDL extravasation occurs not only in late-stage DME, but also in DR, even at very early stage of the disease. Supporting this, in human diabetic retina, we showed that extravascular apoB and ox-LDL were detectable prior to clinical retinopathy (discussed below), suggesting that lipoproteins mediate early pathogenesis of the disease.

## Presence of Modified Lipoproteins in Diabetic Retina of Humans and Animals

To provide evidence that ox-LDL is indeed present in the extracellular space in retinas of DR patients, we recently conducted immunohistochemistry of apoB-100, ox-LDL (antibody against copper-oxidized LDL) and macrophages on the post-mortem retinas from both non-diabetic and type 2 diabetic individuals with varying degrees of DR [61]. Lipoprotein extravasation was observed in all diabetic patients, with the extent correlating with the severity of retinopathy (i.e. diabetic without clinical DR < non-PDR < PDR), but was entirely absent in non-diabetic controls. The finding of ox-LDL in diabetic retinal tissue prior to the onset of clinical DR is consistent with its role in promotion of early DR. Ox-LDL first appeared in the inner retina (i.e. ganglion cell layer) where most blood flow is from the central retinal artery, and permeated later to the outer retina that receives the choroidal circulation. In addition, macrophage infiltration was prominent in retinal sections from patients with PDR. These changes were also accompanied with Terminal-dUTP-Nick-End-Labeling (TUNEL) positive cells in retinas from the diabetic patients, but absent in those from non-diabetic subjects, suggesting cytotoxicity by modified LDL in promoting DR. The data were in line with an earlier case report showing the presence of extravascular apoB, cholesteryl ester and macrophages in retinas obtained from two patients with diabetic maculopathy [49].

Intra-retinal modified LDL has also been observed in a diabetic animal model. Using Akita mice, a well-established model for DR, we detected marked increase of both oxidized and glycated LDL in retina at 13 weeks of age, as compared with wild-type controls; the immunostaining intensity was attenuated following anti-oxidant treatment [62]. It is notable that the timing of our detection of extravasated modified LDL was probably at the early stage of vascular permeability changes in this mouse model, consistent with our findings in humans. Barber et al., also using Akita mice, found increased retinal vascular permeability after 12 weeks of hyperglycemia (~16 weeks of age), but changes of morphology (reduction in the thickness of inner plexiform and nuclear layers, and reduction in the number of cell bodies in the ganglion cell layer) occurred later, after 22 weeks of hyperglycemia, and acellular capillaries and altered morphology of astrocytes and microglia occurred only after 36 weeks of hyperglycemia [63]. Han et al. reported that the early signs of vascular damage (pericyte ghosts, vascular leakage, and microaneurysm formation) appeared at a later stage, approximately 4 months after hyperglycemia, followed by neovascularization 7 months after hyperglycemia [64].

## Effects and Mechanisms of Action of Modified LDL on Retinal Cells

### Retinal capillary cells

We have accumulated considerable evidence of injurious effects of modified LDL towards a variety of retinal cell types *in vitro*. Since capillary damage, and especially pericyte loss, represents one of the earliest pathological features of DR [65,66], extensive efforts have been made to define the effects of modified lipoproteins on retinal vascular cells, although it is recognized that even early DR could involve a broader neurovascular insult [58]. LDL was obtained from healthy donors and modified *ex vivo* to simulate the various degrees of glycation and/or oxidation that occur in diabetes [67,68]. We first tested mildly modified forms of human LDL on bovine retinal capillary endothelial cells and pericytes [67], with the intent of determining whether mild glycation and/or oxidation of LDL occurring in the circulation [29] might contribute to the initiation of retinal capillary injury. We found reduced survival of both cell types upon exposure to low levels of modified LDL, and that toxicity increased in the following order: normal < glycated ≤ minimally oxidized < glycoxidized LDL [67]. The non-modified, native LDL was ineffective in causing cellular damage, suggesting that higher levels of plasma LDL *per se* do not cause injury to retinal vasculature unless modified under diabetic conditions.

Realizing that extravasated, sequestered lipoproteins experience more extensive modification [29], by both oxidation and glycation, than that which occurs in plasma, we have employed LDL preparations with higher degrees of modification in recent studies. The “highly oxidized, glycated” LDL (HOG-LDL) was prepared by copper oxidization, which generates epitopes on LDL similar to those found in humans [29,61]. The modified LDL was applied to cells typically at concentrations ranging up to approximately 30% of plasma LDL level, which we considered physiologically conservative since the tissue levels of ox-LDL are actually considerably higher than in plasma. Thus in atherosclerosis, ox-LDL concentration may be as much as 70-fold higher than in plasma [31]; and since plasma has ample antioxidant capacity, it is possible that most circulating ox-LDL may originate via ‘reflux’ from plaques [69]. The measures of intra-mural ox-LDL concentrations typically represent average values, and may therefore be misleading: for a substance that is non-uniformly distributed, local concentrations at points of retinal vascular leakage or in arterial plaque could be much higher. Such localized LDL leakage and aggregation are reflected by the patchy distribution of apoB and ox-LDL staining in human diabetic retina [61].

When exposed to HOG-LDL, cultured human retinal pericytes experienced significant toxicity, via caspase-dependent apoptosis, in a dose- and time-related fashion [61,62,70-73]. HOG-LDL also appeared to induce autophagy in pericytes, which may represent an alternative cell fate under oxidative stress [72,74]. Several mechanisms including oxidative stress, endoplasmic reticulum (ER) stress, inflammation, and apoptosis have been explored in detail. Oxidative stress has long been considered an initiating factor in diabetic complications and DR [75]. In pericytes, HOG-LDL increased intracellular reactive oxygen species, peroxynitrite (ONOO-), inducible nitric oxide synthase, nitric oxide, as well as 3-nitrotyrosine levels, but depleted the level of glutathione peroxidase 1; these findings are indicative of both oxidative and nitrosative stresses [72,76]. Modification of LDL after α-tocopherol enrichment [77], or in the presence of aminoguanidine [73], abolished the adverse effects of glycated, oxidized, and glycoxidized LDL on bovine retinal endothelial cell and pericyte survival and other endpoints. In the retina from diabetic rats, we detected significantly elevated levels of 4-hydroxynonenal (4-HNE) and 3-nitrotyrosine compared with non-diabetic rats [78]. With regard to the nitrosative stress, we have described at least one affected pathway that may contribute to pericyte apoptosis. In both human retinal pericyte culture and the retina of Akita diabetic mice, HOG-LDL induced tyrosine nitration of prostacyclin synthase and decreased its activity, resulting in thromboxane receptor stimulation which subsequently mediated pericyte apoptosis [62]. The apoptosis was attenuated by inhibition of the thromboxane receptor or cyclooxygenase-2, and also by restoration of the prostacyclin synthase activity with superoxide dismutase or L-N(G)-nitroarginine methyl ester (L-NAME, a nonselective nitric oxide synthase inhibitor) [62]. It has been reported that ox-LDL, but not native LDL, markedly increased lipid peroxidation, cytosolic phospholipase A2 (cPLA2) activation, and arachidonic acid release in a time- and dose-dependent manner in retinal pericytes; these effects were strongly inhibited by cPLA2 inhibition, and by α-tocopherol [79].

Lipid peroxidation, phospholipase A2 activation, and modulation of the downstream eicosanoids represent a classic link between oxidative stress and inflammatory responses. Similar to its effects in pericytes, ox-LDL also activated cPLA2 and released arachidonic acid in both macrophages and fibroblasts; loss of cPLA2 activity, either by genetic knockout in mice, or by treatment with a cPLA2 inhibitor, resulted in attenuation of arachidonic acid release and apoptosis in response to ox-LDL [80]. In parallel findings using rat renal mesangial cells, activation of cPLA2 by ox-LDL resulted in prostaglandin E2 production, which was suppressed by α-tocopherol [81]. In addition, ox-LDL induced cyclooxygenase-2 protein expression and prostaglandin E2 release in endothelial cells [82], consistent with a higher expression of cyclooxygenase-2 in human diabetic retina [83]. Nonsteroidal anti-inflammatory drugs including selective cyclooxygenase-2 inhibitors were beneficial in experimental DR [84], and also showed promise in reducing fluorescein leakage in a small pilot clinical study [85], although that study failed to demonstrate significant benefits in visual function in patients with DME. In our earlier gene array studies we observed altered gene expression of prostaglandin E synthase in human retinal pericytes after exposure to HOG-LDL, but the level of prostaglandin E2 was not measured in that study [68]. These data highlight the importance of the eicosanoid pathway in mediation of ox-LDL-induced inflammation in retinal vasculature.

Some other cellular markers of inflammation have been evaluated. HOG-LDL increased the monocyte chemoattractant protein-1 (MCP-1) secretion, and nuclear factor-κB (NF-κB) activation in human retinal pericytes; the effects were attenuated by pigment epithelium-derived factor (PEDF) in a dose-dependent manner, suggesting that the inhibitory effect of PEDF on MCP-1 was at least partially through the blockage of NF-κB activation [76]. HOG-LDL also selectively reduced the expression of tissue inhibitor of metalloproteinase-3 (TIMP-3) at both mRNA and protein levels in pericytes, a unique effect amongst all other matrix metalloproteinases (MMPs) and their natural inhibitors (TIMPs) [86]. Additional evidence that HOG-LDL induces inflammation in pericytes includes up-regulation of the acute-phase gene, pentraxin 3 [68], which was also up-regulated by ox-LDL in human vascular smooth muscle cells [87] and strongly expressed in atherosclerotic lesions [88].

ER stress has been a newly discovered mechanism that is implicated in DR, and can be induced by ox-LDL [72,89-92]. When incubated with HOG-LDL, human retinal pericytes exhibited eIF2α phosphorylation, ATF6 nuclear translocation, and increased GRP78, typical signs of ER stress [72]. HOG-LDL also increased the expression of sXBP-1 (a transcription factor involved in ER stress), CHOP (an ER specific proapoptotic factor), and other pro-apoptotic factors including caspase-3 and BAX, but decreased the anti-apoptotic protein BCL-2. These data suggest that HOG-LDL induces ER stress and CHOP activation in pericytes, resulting in transcription of a series of pro-apoptotic genes and suppression of BCL-2, eventually leading to apoptosis [72]. ER stress markers were elevated in the retina of a mouse model of combined diabetes and hypercholesterolemia, compared with that of either diabetes or hyperlipidemia alone [72], and were also detectable in the retina of diabetic patients, but not in non-diabetic individuals [72].

To explore the additional mechanisms underlying the apoptosis induced by modified LDL, we investigated the mitogen-activated protein kinase (MAPK) pathway [71]. Exposure to HOG- vs. N-LDL induced similar degrees of phosphorylation of extracellular signal-regulated kinase (ERK), p38, and Jun N-terminal kinase (JNK), and inhibition of ERK, p38, and JNK phosphorylation did not attenuate apoptosis, suggesting that modified LDL elicits apoptosis independent of the MAPK pathway [71]. Recently, we reported evidence supporting a role of the Wnt signaling pathway in DR [78,93]. In retinas from patients with DR and diabetic animals, we detected elevated levels and nuclear translocation of β-catenin, a key effector in the canonical Wnt pathway, together with higher levels of LDL receptor-related proteins 5 and 6, co-receptors of Wnts. Activation of β-catenin by high glucose was attenuated by aminoguanidine, indicative of a role of oxidative stress in the Wnt pathway activation. Consistent with this, Dickkopf homolog 1, a specific inhibitor of the Wnt pathway, ameliorated retinal inflammation and vascular leakage in streptozotocin-diabetic rats, and neovascularization in an oxygen-induced-retinopathy model [93]. In a more recent study, 4-HNE, an important component of ox-LDL, activated the Wnt pathway in retinal endothelial cells and retinal pigment epithelial (RPE) cells; the effect was blocked by the antioxidant, n-acetylcysteine (NAC) [78]. In streptozotocin-diabetic rats, NAC treatment reduced 4-HNE and 3-nitrotyrosine levels, and attenuated the Wnt pathway activity in retina [78].

To gain a panoramic view of the gene expression in pericytes in response to modified lipoproteins, we conducted a microarray study, in which human retinal perictyes were incubated with HOG-LDL vs. glycated LDL vs. native LDL [68]. HOG-LDL induced a gene expression pattern that was markedly distinct from that of N-LDL or G-LDL, whereas the latter two shared a similar expression pattern. A comparison of the responses to HOG- relative to N-LDL revealed 60 genes with differential expression over 1.7 fold in quadruplicate experiments. The HOG-LDL-responsive genes represented members of multiple functional pathways, including fatty acid, eicosanoid, and cholesterol metabolism, fibrinolytic regulation, cell growth and proliferation, cell stress responses, the kinin system, and angiogenesis. These data will help delineate the signalling pathways responsive to modified LDL in pericytes.

### Non-vascular retinal cells

We have recently examined the effects of HOG-LDL on cultured human retinal Müller cells, measuring cell viability, oxidative stress, and ER stress [91]. HOG-LDL reduced cell viability by triggering apoptosis, as shown by increased TUNEL staining, higher levels of cleaved PARP and caspase-3, as well as altering the balance between BAX and BCL-2 in favor of apoptosis. HOG-LDL enhanced both oxidative and ER stresses in Müller cells; and inhibition of either of these stresses attenuated apoptosis. Further, inhibition of oxidative stress by NAC resulted in reduced ER stress, suggesting that the latter is downstream of the former. The effects of HOG-LDL were largely mimicked by 7-ketocholesterol and 4-HNE, two major components of modified LDL.

Recently, we explored the effect of HOG-LDL on human RPE cells [94]. As in other cells, HOG-LDL induced ER stress and reduced the viability in RPEs. Both apoptosis and autophagy contributed to the cell death. We further tested the potential beneficial effects of HDL on HOG-LDL-induced toxicity: native HDL, but not oxidized or glycated HDL, protected RPEs from the insult of HOG-LDL, suggesting that loss of HDL protection due to modification by oxidation and/or glycation in diabetes may represent another mechanism contributing to DR development.

The anatomical positioning of the RPE layer enables the numerous functions of these cells to support the neural retina. These include formation of the outer BRB, supply and exchange of nutrients between retina and choroidal vasculature, retinoid storage and metabolism, maintenance of photoreceptor outer segment length, secretion of growth factors, and many others [95]. Dysfunction of the RPE is recognized in age-related macular degeneration, and has increasingly been implicated in DR [96]. As part of its role in retinal lipid metabolism, the RPE internalizes LDL and ox-LDL in large quantities, via the LDL receptor and CD36 scavenger receptor, respectively [97,98]. Consistent with our findings, earlier studies have shown that ox-LDL impairs processing of outer rod and cone segments by the RPE by perturbing the fusion of lysosomes with phagosomes [99,100], thus accelerating the onset of RPE senescence and death [57,101-103], increasing VEGF and decreasing PEDF expression [57,102,104], impairing outer BRB integrity [57], and enhancing oxidative stress and inflammation [57]. In RPE cells, β-catenin was also elevated by ox-LDL [57], similar to the effect observed in retinal pericytes [78,93]. Overall, the data indicate that extravasated, modified LDL is injurious to retinal cell types beyond the capillary vascular cells, and thus may contribute to the generalized pathology in DR.

## Summary

In conclusion, we propose a hypothesis that serves to unify the data from epidemiological studies, recent clinical trials with fenofibrate intervention, and exploratory laboratory work. In this hypothesis, lipoproteins in the circulation have an indirect, yet important, role in the development of DR, which is contingent on BRB impairment and lipoprotein extravasation, patchy at first, but later widespread. Extravasated lipoproteins become modified by oxidation and glycation, subsequently contributing to prolonged, widespread retinal neurovascular injuries. Additional studies are ongoing to characterize the detailed mechanisms of lipoprotein-mediated retinal injuries: it is hoped that these will offer deeper insights into the DR pathogenesis, and will lead to new measures for prevention and therapy.

## Abbreviations

ACCORD: Action to Control Cardiovascular Risk in Diabetes
Apo: Apolipoprotein
ATF6: Activating Transcription Factor 6
BAX: B-cell Lymphoma 2-Associated X Protein
BCL-2: B-Cell Lymphoma 2
BRB: Blood Retina Barrier
CHOP: C/EBP-Homologous Protein
cPLA2: Cytosolic Phospholipase A2
DCCT: Diabetes Control and Complications Trial
EDIC: Epidemiology of Diabetes Interventions and Complications
DME: Diabetic Macular Edema
DR: Diabetic Retinopathy
ER: Endoplasmic Reticulum
ERK: Extracellular Signal-Regulated Kinase
ETDRS: Early Treatment Diabetic Retinopathy Study
FIELD: Fenofibrate Intervention and Event Lowering in Diabetes
GRP78: 78 kDa Glucose-Regulated Protein
HDL: High-Density Lipoprotein
4-HNE: 4-Hydroxynonenal
HOG-LDL: Highly Oxidized, Glycated LDL
JNK: Jun N-terminal Kinase
LDL: Low-Density Lipoprotein
L-NAME: L-N(G)-nitroarginine Methyl Ester
LOX-1: Lectin-like ox-LDL Receptor 1
MAPK: Mitogen-Activated Protein Kinase
MCP-1: Monocyte Chemoattractant Protein-1
MMP: Matrix Metalloproteinase
NF-κB: Nuclear Factor-κB
N-LDL: Native LDL
NMR-LSP: Nuclear Magnetic Resonance Lipoprotein Subclass Profile
NPDR: Non-proliferative Diabetic Retinopathy
Ox-LDL: Oxidized LDL
PARP: Cleaved Poly ADP Ribose Polymerase
PEDF: Pigment Epithelium-Derived Factor
PPAR: Peroxisome Proliferator Activated Receptors
p-eIF2α: Phospho-eukaryotic Initiation Factor 2α
RPE: Retinal Pigment Epithelium
sXBP1: Spliced X-box Binding Protein 1
TIMP-3: Tissue Inhibitor of Metalloproteinase-3
TUNEL: Terminal Deoxynucleotidyl Transferase dUTP Nick End Labelling
VEGF: Vascular Endothelial Growth Factor
